# High-throughput analysis of insecticides on malaria vectors using liquid chromatography tandem mass spectrometry

**DOI:** 10.1371/journal.pone.0211064

**Published:** 2019-02-07

**Authors:** Astrid Spielmeyer, Marc F. Schetelig, Josiane Etang

**Affiliations:** 1 Justus Liebig University Giessen, Institute for Food Chemistry and Food Biotechnology, Giessen, Germany; 2 Justus Liebig University Giessen, Institute for Insect Biotechnology, Giessen, Germany; 3 Organisation de Coordination pour la lutte contre les Endémies en Afrique Centrale (OCEAC), Institut de Recherche de Yaoundé (IRY), Yaoundé, Cameroun; 4 University of Douala, Faculty of Medicine and Pharmaceutical Sciences, Douala, Cameroon; Universidade Federal do Rio de Janeiro, BRAZIL

## Abstract

**Background:**

Different setups and protocols have been developed for investigating insecticide effects on *Anopheles* (*An*.) mosquitoes, vectors of malaria. However, chemical uptake resulting from their tarsal contact with insecticide-treated material has seldom been investigated. To address the challenges encountered in the interpretation of bioassay data, a high throughput method for chemical analysis on malaria vectors was developed and validated for five selected insecticides including alpha-cypermethrin (aCYP), deltamethrin (DM), etofenprox (EPX), permethrin (PM), pirimiphos-methyl (PPM).

**Methods:**

The method includes a single chemical extraction step via an ultrasound probe on mosquito samples and analysis via liquid chromatography coupled to high-resolution tandem mass spectrometry (UHPLC-MS/MS). The protocol was established for two malaria vector species, *Anopheles gambiae senso stricto* (*s*.*s*.) and *An*. *stephensi*, both males and females. Recovery rates ranged from 70 to 100% without any influence of sex or species. The method was efficiently applied to female *An*. *gambiae s*.*s*. of the KISUMU1 reference strain, after susceptibility tests using the World Health Organization’s standard protocol.

**Results:**

Susceptibility tests revealed 13.4–18.4 minutes knockdown times for 50% mosquitoes during exposure to EPX and pyrethroids. The mortality rates 24 hours post-exposure to insecticides were mostly 99–100%, except in two PM and three PPM assays suggesting possible or confirmed resistance to these insecticides. The mean insecticide uptake in dead mosquitoes ranged from 23 pg (aCYP) to 1812 pg (EPX) per specimen. However, the mean uptake in survivors to PM and PPM was reduced by at least 25%, suggesting that acute doses were not achieved in these specimens during bioassays.

**Conclusions:**

The developed and validated UHPLC-MS/MS method could be used to address some limitations of bioassays or to assess the penetration of insecticides in mosquito matrix with reference to cuticle thickness and other insecticide resistance mechanisms.

## Introduction

Malaria remains one of the most deadly diseases in the world. In 2016, an estimated 216 million cases were recorded, leading to 445.000 deaths, mainly in children aged less than five years in Africa (92%) [1]. Since 2000, 663 million clinical cases have been averted by Insecticide Treated Nets (ITNs) and Indoor Residual Spraying (IRS) (jointly 78%) as well as Artemisinin Combination Therapies (ACTs) (22%) [2]. In few specific settings and circumstances, ITNs and IRS are supplemented by larval source management including larviciding, environmental measures or improvement of the housing to reduce the suitability of the environment as mosquito habitat or to restrict the human biting rates [3, 4]. This emphasizes the need for continued investments in malaria vector control, aiming at shortening the lifespan of mosquitoes near their human targets [5, 6]. Attempts to develop specific assays and new technologies that would sustain interventions against malaria or prompt the identification of public health insecticides with novel modes of action are therefore of high interest.

Due to their cost effectiveness and operational flexibility, ITNs are widely used for malaria prevention in endemic countries [2]. They have a dual mode of action, namely a physical barrier preventing the human-mosquito contact and a chemical barrier provided by the insecticide against host-seeking mosquitoes that come into contact with the net. The efficiency of ITNs and IRS greatly depends on insecticidal properties including spatial repellency/ deterrence, contact irritancy, knockdown (KD) and killing effects as well as inhibition of life traits of local anopheline species [7–10]. A number of methods have been developed to assess insecticide toxicity on mosquitoes after topical application or tarsal contact with treated substrates, in order to screen new compounds, evaluate the efficacy of chemicals or to assess insect resistance to insecticides. Among those, the most common tests are dose-response or discriminating dosage bioassays [11–14]. Although these bioassays provide data concerning the toxic activity of compounds on target mosquitoes, there is a gap of knowledge on the accurate measurements of insecticide uptake by the insect tarsus. This uptake by the mosquitoes after contact, e.g. with vapor, particles or surface residues is essential as it will determine whether or not the mosquitoes pick up a lethal dose before escape [15–17]. Therefore, measurements of mosquito phenotypic response to chemicals may be supplemented by new tools to characterize the activity of insecticides and the impact of vector control programs on mosquito populations.

Currently, the shortage of methods to determine the minimum chemical amount sufficient to kill the targeted mosquitoes sometimes weakens the accuracy of insecticide testing protocols. The chromatographic analysis of insecticides mainly focusses on the residue analysis in food matrices, and both gas and liquid chromatography usually coupled to (tandem) mass spectrometry are utilized [18–21]. Methods related to malaria prevention deal, e.g., with the analysis of insecticides on ITNs for quality control or with monitoring their fate over time [22–24]. The aim of this study was the development and validation of an analytical method for the determination of five selected insecticides in mosquito samples using liquid chromatography and time of flight (ToF) tandem mass spectrometry (UHPLC-MS/MS). The method was applied to female *Anopheles (An*.*) gambiae sensu stricto* (*s*.*s*.), the primary malaria vector in Africa, after exposure to insecticides using the World Health Organization’s (WHO) susceptibility test. Within the European Union (EU) guidelines for sampling, sample analysis, and data evaluation are provided by SANTE/11813/2017 [25]. This guidance document aims at a harmonized analysis of pesticides in food and feed, but can also be utilized as a reference for other pesticide analysis methods.

## Material and methods

### Reagents and materials

Acetonitrile (UHLC/MS grade) was purchased from ActuAll (Oss, Netherlands). Standards of the investigated insecticides alpha-cypermethrin (aCYP), deltamethrin (DM), etofenprox (EPX), permethrin (PM) and pirimiphos-methyl (PPM) as well as the internal standards D_5_-aCYP, D_5_-DM, D_5_-PM and pirimiphos-ethyl (PPE) were purchased from Sigma-Aldrich (Taufkirchen, Germany) in PESTANAL purity. The selected insecticides are recommended and commonly used in long-lasting insecticidal nets (LLINs) or IRS.

Method development and validation were performed using non-treated mosquitoes of two known malaria vector species, namely *An*. *gambiae s*.*s*. and *An*. *stephensi*. The Kisumu laboratory reference strain of *An*. *gambiae s*.*s*. isolated from Kenya in 1975 (VectorBase, http://www.vectorbase.org, KISUMU1) is known to be free of any detectable insecticide resistance mechanism and was kindly provided by Dr. Pie Müller from the Swiss Tropical Institute (Basel, Switzerland). *An*. *stephensi* is a major malaria vector from the Middle East through the Indian subcontinent and China (https://www.vectorbase.org/organisms/anopheles-stephensi). Mosquitoes used for this study belong to the SD500 strain obtained from Dr. Andrew M. Blagborough, Imperial College London (London, UK). Both *Anopheles* strains were reared in standard conditions (27±3 °C, 60–80% relative humidity, 12:12 light-dark cycle). Larvae were fed with TetraMin Baby fish food. Adult mosquito diet was 10% sucrose with adult females fed twice a week with cow or pig blood that was purchased from a certified butcher shop. The experiments conducted do not need an ethics committee approval.

For method validation, male and female mosquitoes were treated separately using 75–120 mosquitoes per sample. The weight of the samples varied between 40 and 108 mg (male) respective 96 and 150 mg (female). Mosquitoes were transferred to 15 mL PP centrifuge tubes, weighed and stored at -20 °C before further treatment.

For mosquito bioassays, five diagnostic concentrations (DC) of insecticides coated on Whatman n°1 filter paper sheets (12x15 cm) were supplied by the Vector Control Research Unit (VCRU) of University Sains Malaysia (Penang, Malaysia). Filter papers were impregnated with technical-grade insecticides or control solution (acetone and silicon oil), shipped from Malaysia and kept at 4 °C until usage. Acetone was utilized as the solvent, whereas silicon oil served as the carrier for insecticides. The applied DC were as follows: 0.05% aCYP, 0.05% DM, 0.75% PM (all pyrethroids), 0.5% EPX (pseudo pyrethroid, pyrethroid ether), 0.25% PPM (organophosphate).

### Sample preparation

**Fig 1:**
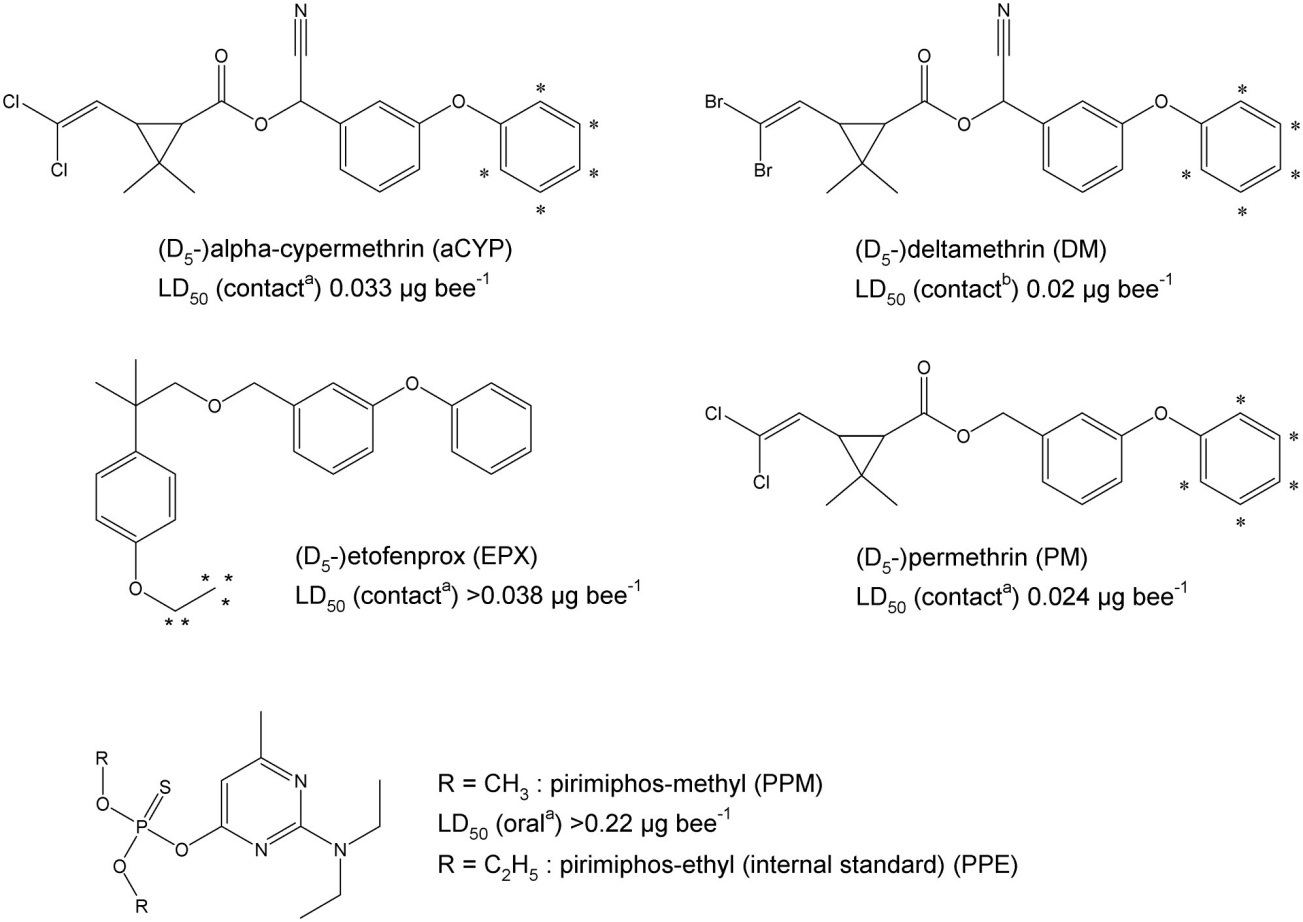
Structures of insecticides investigated. *—position of deuterium; LD_50_ values refer to honey bees and 48 h exposure in case of contact toxicity, values were taken from [26] (a) respective [27] (b).

The structures of insecticides selected for the development of LC-MS/MS are presented in Fig 1. Mosquito samples were directly prepared in the centrifuge tube used for storage. For the insecticide extraction, 50 μL of a mix internal standard solution was added resulting in 20 ng (PPE, D_5_-EPX) respective 100 ng (D_5_-aCYP, -DM, -PM) of the compounds on the mosquitoes. Afterwards, the non-labelled analytes were added as a methanolic solution (method validation) and/or methanol was added to reach a final volume of 1 mL. The extraction was performed using an ultrasound probe (MS72, Bandelin, Berlin, Germany). Each sample was treated three times for 15 sec using a cycle time of 0.5 sec and a power setting of 50% (Bandelin Sonopuls HD2070). Samples were cooled during and after ultrasound treatment using ice water to avoid analyte loss. Extracts were centrifuged (1000 g, 3 min) and the supernatant was transferred into a 1.5 mL PP centrifuge tube (Eppendorf). This solution was centrifuged again (17.000 g, 3 min) and utilized for analysis. Extracts were stored at -20 °C. If samples revealed an analyte concentration below the respective quantification limit, 200 μL of the extract were concentrated in a stream of nitrogen to approx. 30 μL.

### LC-MS/MS analysis

Chromatographic separation was performed using a Nexera X2 UHPLC (Shimadzu, Duisburg, Germany) equipped with an Accucore RP-MS column (2.6 μm, 2.1 mm x 100 mm, Thermo Scientific). For detection, a TripleTOF 5600+ system (AB Sciex, Darmstadt, Germany) was utilized. After every ninth sample, the system was calibrated via the Calibrant Delivery System using sodium formiate [28]. The eluent flow was split before entering the mass spectrometer interface (1:2).

Gradient elution was performed using 5 mM ammonium acetate containing 1% acetonitrile (solvent A) and acetonitrile (solvent B). The flow rate was set to 0.30 mL min^-1^, the column was kept at room temperature (22 °C). Separation started with 70% B and this portion was increased to 90% within 6 min. The ratio was set back to 70% B within 0.5 min and this setting was kept for 1.5 min resulting in an overall analysis time of 8 min per run. For both standards and sample solutions, 2 μL were injected using a sample loop.

For detection, both full scan and product ion scans were recorded. A full scan was performed from 50 to 700 m/z, for product ion scans the respective quasi molecular ion [M+H]^+^ (PPM, PPE) or ammonium adduct [M+NH_4_]^+^ was selected and the range from 50 to 500 m/z was recorded (Table 1). A mass window of ± 3 mDa was used for extraction corresponding to a mass accuracy of less than 10 ppm for the full scan mode and less than 17 ppm (28 ppm for PPM) for the product ion scan mode. The following parameters were utilized for all analyses: ion spray voltage 5500 V, interface temperature 400 °C, accumulation time 100 msec, collision gas nitrogen.

**Table 1:** Fragment and molecular ions utilized for substance quantification and qualification with their exact masses and molecular formulas.

Substance	quantifier ion [*m/z*]	qualifier ion [*m/z*]	CE^a^ [V]	retention time [min]
	molecular formula	molecular formula		
aCYP	191.0025	433.1080^b^	20	4.11
	C_8_H_9_Cl_2_O^+^	C_22_H_23_Cl_2_N_2_O_3_^+^		
D_5_-aCYP	191.0025	438.1394^b^	20	3.95/4.10
	C_8_H_9_Cl_2_O^+^	C_22_H_18_D_5_Cl_2_N_2_O_3_^+^		
DM	278.9015	521.0070^b^	20	4.22
	C_8_H_9_Br_2_O^+^	C_22_H_23_Br_2_N_2_O_3_^+^		
D_5_-DM	278.9015	526.0384^b^	20	4.18
	C_8_H_9_Br_2_O^+^	C_22_H_18_D_5_Br_2_N_2_O_3_^+^		
EPX^c^^,^^d^	177.1274	183.0804	30	4.87
	C_12_H_17_O^+^	C_13_H_11_O^+^		
D_5_-EPX^c^^,^^d^	182.1588	183.0804	30	4.82
	C_12_H_12_D_5_O^+^	C_13_H_11_O^+^		
PM	183.0804	408.1128^b^	20	4.59/4.99
	C_13_H_11_O^+^	C_21_H_24_Cl_2_O_3_N^+^		
D_5_-PM	188.1118	413.1442^b^	20	4.55
	C_13_H_6_D_5_O^+^	C_21_H_19_D_5_Cl_2_O_3_N^+^		
PPE	198.1059	182.1288	35	3.17
	C_9_H_16_N_3_S^+^	C_9_H_16_N_3_O^+^		
PPM^c^	108.0556	164.1182	35	2.28
	C_5_H_6_N_3_^+^	C_9_H_14_N_3_^+^		

^a^ collision energy,

^b^ detected as ammonium adduct,

^c^ quantification and qualification via product ions due to matrix interferences in the full scan chromatogram,

^d^ selection of ammonium adduct as precursor ion;

aCYP—alpha-cypermethrin, DM—deltamethrin, EPX—etofenprox, PM—permethrin, PPE—pirimiphos-ethyl, PPM—pirimiphos-methyl.

### Method validation

Blank samples of both *Anopheles* species, including females and males, were prepared as described above without the addition of the internal standards. Both full scan and product ion scans chromatograms were checked for potential matrix interferences.

Stock solutions (1 mg mL^-1^) were prepared in methanol and were stored in the dark at -20 °C. Mosquito extracts were prepared with absolute masses of 0.5, 2.5, 50, 250 ng (PPM, EPX) respective 15, 50, 250 ng (aCYP, DM, PM) on the mosquitoes (single determination for males and females of each species, n = 4 per concentration level). Samples were prepared as described above and stored at -20 °C. The extracts were immediately analyzed as well as after 1, 3, 7, 14 and 28 days to determine sample stability. Measurements were performed as triplicate. Recovery rates were calculated referring to pure methanol standard solutions of the corresponding concentrations (0.5 to 250 μg L^-1^) which were analyzed on day 0.

Samples prepared for stability tests were utilized for further method validation parameters. Values determined on day 0 were used to prove the accuracy of the method. Intra-day and inter-day precision were determined for matrix samples with a content of 50 ng (absolute mass on mosquitoes) respective 50 μg L^-1^ extract concentration by threefold injection. For intra-day precision, samples were analyzed after 0, 6, 11 and 17 h. The method precision was determined for each concentration level by calculating the relative standard deviation (RSD) of the four recovery rates obtained for different species and sexes. Measurement precisions were calculated for each concentration level as triplicate analysis.

The limit of detection (LOD) and limit of quantification (LOQ) were determined by calculating the signal-to-noise ratio (SN) of the extracted ion chromatogram of the full scan or the product ion scan (Table 1). LOD was defined as an SN of 3:1 of both the quantifier and qualifier ion. LOQ was defined as an SN of 10:1 of the quantifier ion.

Due to the small mass window utilized for extraction, the background signals usually were below 6 counts per second (cps) in standard solutions and blank mosquito extracts, and they were absent in many samples. In addition to the SN criteria, a minimum signal intensity of 20 cps and 30 cps was considered for the LOD and LOQ in case of absent background signals. Based on the results obtained for pure standard solutions, pooled blank mosquito extracts were spiked with different concentrations of the investigated insecticides, and the SN was calculated to determine the matrix LOD respective LOQ (mLOD, mLOQ).

**Fig 2:**
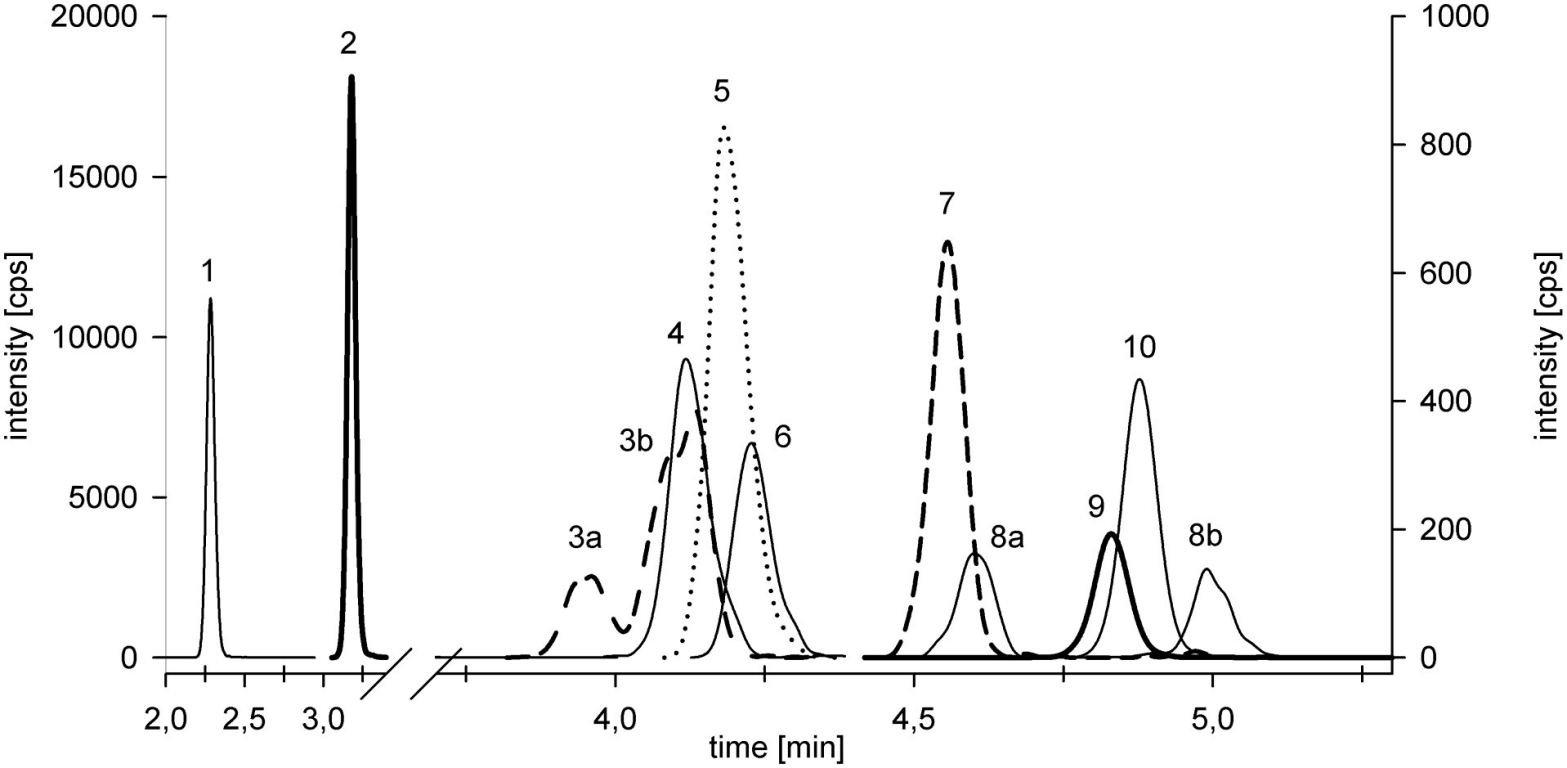
LC-MS/MS chromatogram of a standard solution containing 50 μg L^-1^ of the analytes respective 20 μg L^-1^ (PPE, D_5_-EPX) and 100 μg L^-1^ (D_5_-aCYP, -DM, -PM) of the internal standards. Right y-axis used for aCYP, DM and PM and their deuterated analogues; extracted ion chromatogram of the quantifier ion is shown with 1—PPM, 2—PPE, 3—D_5_-aCYP, 4—aCYP, 5—D_5_-DM, 6—DM, 7—D_5_ PM, 8—PM, 9—D_5_-EPX, 10 EPX.

Linearity was tested for standard solutions with concentrations ranging from the LOQ up to 500 μg L^-1^ containing the internal standards with 20 μg L^-1^ (PPE, D_5_-EPX) and 100 μg L^-1^ (D_5_-aCYP, -DM, -PM). The area ratio of the quantifier ion of the analyte and the corresponding internal standard was plotted relative to the analyte concentration (μg L^-1^) (Fig 2).

### Bioassays

**Fig 3:**
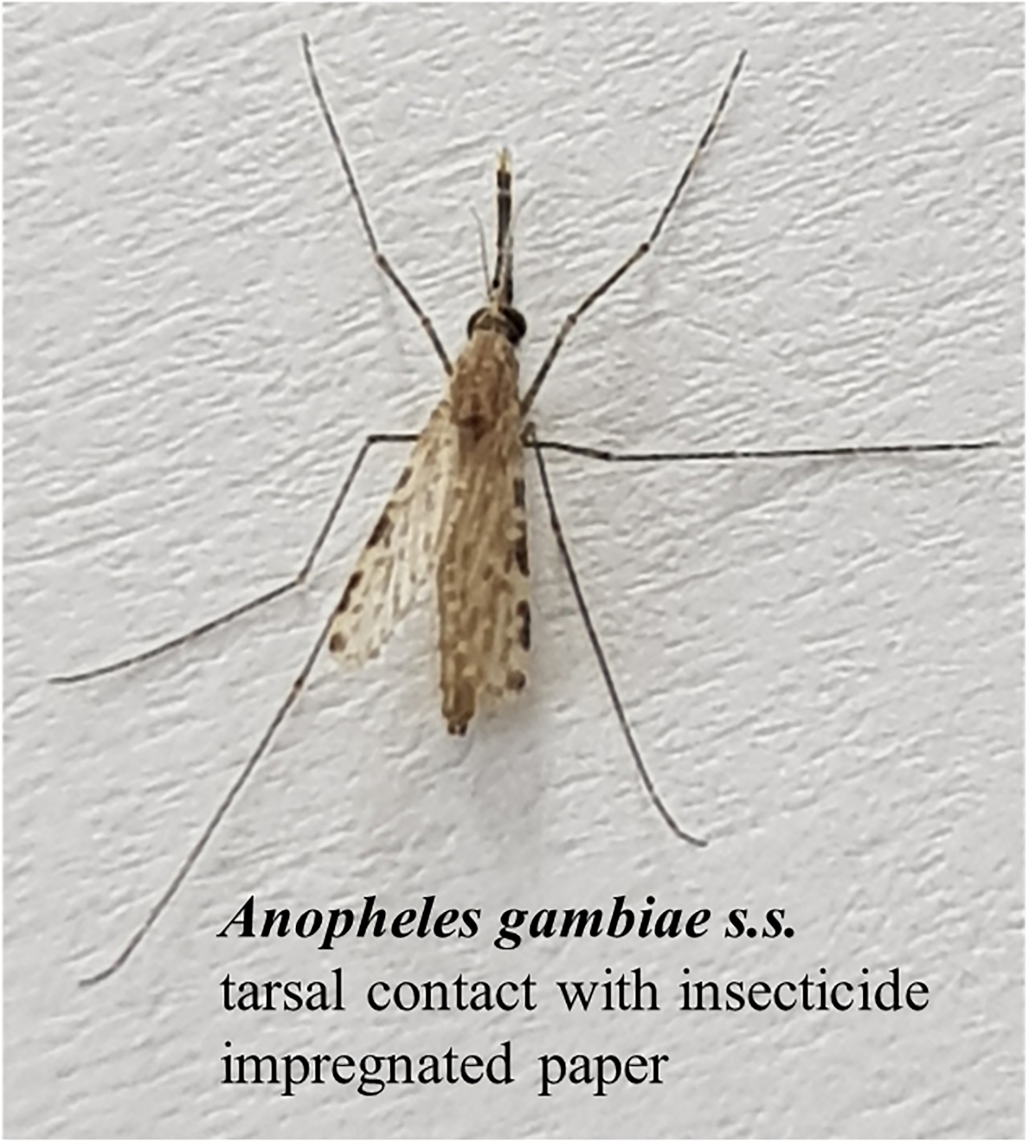
Picture of *Anopheles gambiae s*.*s*. tarsal contact with insecticide impregnated paper during susceptibility test.

Bioassays were performed on 2–4 days old unfed female mosquitoes of *An*. *gambiae s*.*s*. under ambient room temperature (25–27 °C) and relative humidity (70–80%), using WHO susceptibility test kits and the standard protocol for adult mosquitoes [29]. Each full set of bioassays was performed with five to six batches of 20–25 females. Four batches were exposed to insecticide impregnated filter papers (Fig 3) and one to two batches were exposed to untreated filter papers as the control. During exposure, the number of mosquitoes knocked down (i.e. unable to maintain normal posture or to fly—falling to the bottom of the test tube) was recorded at 5 min intervals. After 1 h exposure, mosquitoes were transferred into holding tubes, provided with cotton pads soaked with 10% sugar solution and kept in the incubator (27 °C, 70% humidity). The mortality rates were determined 24 h post exposure (susceptibility tests). Then, mosquito samples were pooled in 15 mL PP centrifuge tubes, weighed and kept at -20 °C before further treatment. Control mosquitoes, as well as survivors of the respective assays, were immobilized for 15 min at -20 °C, before being pooled and kept separately.

Mosquito samples after tarsal contact bioassays with diagnostic concentrations of insecticides were subject to chemical analysis using UHPLC-MS/MS as described in the preceding sections.

### Data analysis

Data on mosquito susceptibility to insecticides were analyzed according to the WHO criteria. Mortality rates <90% indicate a resistance whereas mortality rates of 90–97% refer to a possible resistance which has to be confirmed in a repeated test. Mortality rates equal to or higher than 98% indicate susceptibility of the tested samples [29]. The knockdown rates were subjected to a computerized log-probit analysis using WIN DL software (version 2.0, 1999) to determine the KD times for 50% of the mosquitoes within one set (KdT_50_) as well as their 95% confidence interval (CI), based on regression lines. Tested mosquitoes were considered as having the same KD times when 1) their probit lines were parallel (parallelism test not rejected at 95% CI), and 2) the ratio between their KdT_50_ had confidence limits including the value 1 [30, 31]. Statistical analysis (t-test) was performed using SigmaPlot (version 12.5).

## Results

### LC-MS method development

The molecular ion [M+NH_4_]^+^ was detected in directly infused standard solutions of pyrethroids (aCYP, DM, PM) and the pseudo pyrethroid EPX which underlines the high affinity of these compounds to the ammonium ion. The protonated molecular ion was less intensive than adducts of sodium, potassium or ammonium. Consequently, ammonium acetate was added to the aqueous eluent to enhance the formation of the ammonium adduct and to improve the LOD. The ionization of the organophosphate PPM was not negatively affected by this additive. Although the ammonium adducts were utilized as precursors for the MS/MS analyses, product ions were detected in their protonated form (Table 1).

For PM and D_5_-aCYP, an isomer mixture was utilized, and both isomers were considered for quantification (Table 1, Figs 1 and 2). In the case of PM, the presence of the two isomers was used as additional criterion for the unequivocal identification of the analyte.

For few samples, the full scan chromatogram revealed minor peaks for the molecular ions of EPX and PPM which were shifted in retention time by approx. 0.1 min. These interferences showed no trend concerning species, sex or mosquito biomass. To avoid false positive results, a second fragment ion was utilized for analyte identification in case of these analytes (Table 1). Blank samples of the investigated mosquitoes did not reveal any signals at the selected fragments or (pseudo) molecular ions of the analytes and internal standards.

### Development of extraction protocol

Ultrasound treatment was found to destroy the mosquito torso efficiently. Three cycles of 15 sec were preferred over one cycle of 45 sec as mosquito torsos were able to sediment in between which enhanced the efficiency of the torso destruction. The ultrasound probe allowed the usage of small liquid volumes (1 mL) for extraction. This way, low mLOD and mLOQ were achieved without further sample treatment (Table 2). Larger extraction volumes were avoided as especially in case of aCYP and DM an extensive reduction of the extract volume would have been necessary to reach a signal-to-noise ratio (SN) higher than 10 in the mosquito extracts (see below).

**Table 2:** Detection and quantification limits of the studied compounds in methanol (LOD, LOQ) and matrix solutions (mLOD, mLOQ).

Substance	LOD	LOQ	mLOD^a^	mLOQ^a^	Linearity range
	[μg L^-1^]	[μg L^-1^]	[μg L^-1^]	[μg L^-1^]	[μg L^-1^]
aCYP	1.0	2.5	2.0	3.0	2.5–500
DM	1.5	5.0	2.0	5.0	5.0–500
EPX	0.1	0.2	0.1	0.2	0.2–350
PM	5.0	7.5	2.0	7.5	7.5–350
PPM	0.025	0.1	0.05	0.1	0.1–350

^a^ values refer to non-concentrated extracts, (m)LOD—(matrix) limit of detection, (m)LOQ—(matrix) limit of quantification, aCYP—alpha-cypermethrin, DM—deltamethrin, EPX—etofenprox, PM—permethrin, PPM—pirimiphos-methyl.

### Validation of the LC-MS/MS method

PPM and EPX showed similar quantification limits which were a least one order of magnitude lower than the limits of the investigated pyrethroids (Table 2). The quantification limits for matrix and matrix-free solutions were similar or identical which implies a negligible effect of the matrix on the ionization of the analytes (Table 2). In the case of the pyrethroids, assay samples showed results below the mLOQ. An aliquot of these extracts was concentrated by approximately a factor of 7 in a stream of nitrogen to reach an SN above 10 to allow a quantification of the insecticides. To avoid substance loss, extracts were not heated but were kept at room temperature during that time. Deuterated compounds can slightly differ in the volatility compared to the non-labelled compound [32]. Thus, the concentration step was limited to 10 min. Linearity was proven according to DIN 38402–51 [33] (Table 2). Concentrations up to 350 respective 500 μg L^-1^ enable sample analyses without prior dilutions over a wide concentration range. Measurement and method precision was highest when close to the mLOQ. The measurement precisions ranged from 1.5 to 9.9%, depending on the concentration and the analyte. Intra-day precision ranged from 1.1 to 6.7%, inter-day precision (excluding PM after 14 days and PPM) from 1.4 to 5.0%. Method precision was below 10% in most of the cases (Table 3).

**Table 3:** Recovery rates determined for *An*. *gambiae* and *An*. *stephensi* depending on extract concentration and storage time.

Substance	Concentration	0 d	1 d	3 d	7 d	14 d	28 d
	[μg L^-1^]	Recovery average ± SD [%]					
aCYP	15	93 ± 10	97 ± 10	93 ± 5	92 ± 7	99 ± 4	92 ± 6
	50	95 ± 1	95 ± 1	100 ± 5	101 ± 3	99 ± 5	95 ± 4
	250	95 ± 4	96 ± 1	98 ± 1	101 ± 2	103 ± 1	102 ± 4
DM	15	93 ± 13	95 ± 11	104 ± 10	102 ± 4	101 ± 8	96 ± 15
	50	94 ± 3	95 ± 3	98 ± 3	98 ± 6	96 ± 3	93 ± 3
	250	95 ± 4	94 ± 2	94 ± 4	96 ± 1	93 ± 3	96 ± 1
EPX	0.5	99 ± 9	114 ± 10	118 ± 20	120 ± 15	125 ± 4	107 ± 8
	2.5	90 ± 2	94 ± 2	94 ± 5	86 ± 2	94 ± 4	86 ± 2
	50	95 ± 2	97 ± 2	97 ± 4	98 ± 5	104 ± 2	94 ± 2
	250	94 ± 4	102 ± 4	103 ± 3	104 ± 3	108 ± 5	94 ± 3
PM	15	68 ± 5	72 ± 7	68 ± 3	67 ± 4	73 ± 4	89 ± 3
	50	87 ± 6	84 ± 3	85 ± 2	84 ± 6	86 ± 5	108 ± 9
	250	81 ± 4	79 ± 5	81 ± 5	78 ± 5	81 ± 2	103 ± 6
PPM	0.5	81 ± 14	135 ± 23	139 ± 8	171 ± 24	188 ± 32	153 ± 20
	2.5	88 ± 8	102 ± 10	112 ± 11	121 ± 10	153 ± 12	135 ± 8
	50	93 ± 7	99 ± 6	110 ± 6	120 ± 7	142 ± 3	134 ± 5
	250	85 ± 11	103 ± 5	116 ± 7	124 ± 5	148 ± 4	137 ± 8

Relative recovery rates refer to average of four samples (two species, two sexes) which were measured as triplicate; SD—standard deviation; aCYP—alpha-cypermethrin; DM—deltamethrin; EPX—etofenprox; PM—permethrin; PPM—pirimiphos-methyl.

On day 0, recovery rates were between 80 and 100% for all analytes and concentrations except low concentrations of PM. Only in this case, a correction of the determined concentration was performed by a factor of 1.2 to obtain results comparable to concentrated PM solutions (quotient of mean recovery rates obtained for high and low concentrated extracts, Tables 3 and 4). Recovery rates lower than 70% are acceptable if the method precision is below 20% [25]. These criteria are fulfilled for all the samples except low concentrations of PPM after storage (Table 3). For those samples, standard deviations of more than 30% were observed. Furthermore, all samples containing PPM showed an increase in the recovery rate with prolonged storage time. This effect was more pronounced for smaller concentrations. The signal intensity of both the analyte PPM and the internal standard PPE decreased within 28 days, but the reduction was more pronounced for PPE.

**Table 4:** Mortality rate, KdT_50_ and insecticide load for *An*. *gambiae* (female) exposed to insecticides within the WHO susceptibility test.

Insecticide DC	# Mosquitoes^a^	Sample weight	Extract concentration	ng per g biomass	pg per mosquito	Mortality rate	KdT_50_ (CI)	# Filter paper usage
		[mg]	[μg L^-1^]			[%]	[min]	
0.05% aCYP	89	66.9	2.1^b^	31	23	100	18.2 (16.5–20.1)	1^st^
	106	45.4	3.1^b^	48	29	100	14.9 (10.9–17.3)	2^nd^
	95	55.1	3.1^b^	60	35	100	15.8 (14.2–17.1)	3^rd^
0.05% DM	101	58.1	5.3^b^	91	52	100	15.7 (12.5–19.4)	1^st^
	82	44.1	2.8^b^	63	34	100	15.3 (10.0–20.4)	2^nd^
	93	53.8	4.2^b^	78	45	100	15.3 (13.7–16.8)	3^rd^
0.5% EPX	105	29.9	190	6358	1812	100	13.6 (11.9–15.2)	2^nd^
	98	58.1	159	5741	1626	100	18.4 (17.7–19.1)	3^rd^
	95	28.7	74	2597	784	100	14.8 (10.8–17.8)	4^th^
0.75% PM	89	47.9	43	898	483	92.1	15.2 (14.5–16.0)	1^st^
	95	37.4	44	1180	464	95.0	14.2 (13.5–14.8)	2^nd^
	96	43.0	102	2368	1061	99.0	13.4 (12.2–13.8)	4^th^
	10^c^	11.4	2.9^b^	257	292	---	---	
				(308)^d^	(351)^d^			
0.25% PPM	62	59.0	n.d.	---	---	1.6	NA	1^st^
	86	78.5	n.d.	---	---	2.3	NA	2^nd^
	78	70.4	n.d.	---	---	5.1	NA	3^rd^
	51	44.0	53^e^	1216	1049	100	NA	1^st^

^a^ if not stated otherwise, numbers refer to dead mosquitoes;

^b^ samples were concentrated to reach SN higher than 10 respective signal height higher than 30 cps,

^c^ survivors of the corresponding sets, separately analyzed;

^d^ values corrected by factor 1.2 due to different recovery rates (see Table 3);

^e^ results from a preliminary test conducted seven months before using the same batch of impregnated filter paper; KdT_50_—knockdown time for 50% of the mosquitoes;

CI—confidence interval; DC—diagnostic concentration; NA—not applicable, n.d.—not detected, aCYP—alpha-cypermethrin; DM—deltamethrin; EPX—etofenprox; PM—permethrin; PPM—pirimiphos-methyl.

Reduced stability of small concentrations was also observed in the case of EPX. Based on these results, samples containing PPM and EPX were measured right after preparation. In the case of PM, an increase in the recovery rate was observed after 4 weeks compared to the previous measurements, so these samples have to be analyzed within 2 weeks after preparation. In case of the other analytes and concentrations, samples could be stored at -20 °C for at least 4 weeks.

Aside from the limitations mentioned above, no influence of the concentration on the recovery rate was observed and constant or proportional systematic errors can be excluded (Table 3). Consequently, calibration and quantification can be conducted using matrix-free solvent standards.

### Knockdown times, mortality rates, weight and chemical uptake in *Anopheles gambiae s*.*s*.

Data from mosquito bioassays and subsequent chemical analyses are presented in Table 4. A total of 2456 female *An*. *gambiae s*.*s*. were used for insecticide susceptibility tests, including 740 control and 1716 test specimens. Each test was repeated three times to assess the reproducibility of the assay, and the corresponding number of impregnated paper utilization was recorded. Impregnated papers were used up to four times. Overall, 16 susceptibility tests were carried out from April 2017 to January 2018.

For chemical analysis, the quantification was performed using an external one point calibration (50 μg L^-1^, diluted in pure methanol) without correction by recovery rates except for PM extracts with concentrations below 20 μg L^-1^. Here a correction by factor 1.2 was performed (see above). The analyte concentration (μg L^-1^) was utilized as criteria for defining results as above or below the mLOQ.

Among the mosquito samples used as a control during the bioassays, knockdown and mortality rates were 0–3%, indicating that the susceptibility tests were properly performed. For mosquito samples exposed to EPX and the pyrethroids, the knockdown times for 50% of the tested individuals (KdT_50_) were 13.4–18.4 minutes (Table 4). Based on overlapping confidence intervals as well as differences of less than factor 1.4 between the respective KdT_50_, no significant difference of KdT_50_ was recorded neither between the triplicate of each insecticide nor between the four tested compounds nor the number of filter paper utilization. For PPM, the knockdown rates during exposure to impregnated paper were lower than 50% (Table 4).

The mortality rates 24 hours after exposure to aCYP, DM and EPX as well as one replicate of PM were 99–100%. In two PM trials, mortality rates of less than 98% were obtained which suggest a probable resistance. For PPM, a mortality rate of 100% was obtained during a preliminary test, but it dropped to less than 6% for the three following replications carried out seven months after that (Table 4).

Following the bioassays, the mean biomass of control mosquitoes was 0.92 (± 0.16) mg per individual which was significantly higher than the weight of individuals treated with insecticides (0.48 ± 0.16 mg) (t-test, *p* < 0.001). However, mosquitoes exposed to PPM showed a weight comparable to control individuals (0.92 ± 0.03 mg per individual, *p* > 0.5).

For insecticide analyses, the triplicates of dead mosquitoes after exposure to aCYP or DM showed comparable profiles; the mean uptake was 29 and 44 pg insecticide per mosquito, respectively (Table 4). These insecticides were utilized with the lowest DC (0.05%), and exposed mosquito samples showed the lowest masses per specimen. These masses were sufficient to kill all treated mosquitoes. PM, another pyrethroid, was applied in a 15 times higher DC (0.75%) than aCYP and DM and PM masses per mosquito were accordingly higher (mean of 670 pg per mosquito). The highest masses per insect were obtained for the pseudo pyrethroid EPX (mean of 1407 pg per mosquito) which was two times higher than the mass of PM, even though applied in a lower DC (0.5%). Although mosquitoes revealed a remarkably lower EPX uptake within the third trial respective fourth filter paper usage, the amount was sufficient to kill all the exposed individuals.

The survivors (n = 10) resulting from exposure to PM were mainly collected during the first and second filter paper usage, and they were separately analyzed. The average chemical uptake per specimen was 25% reduced compared with dead mosquitoes (Table 4). For mosquitoes being exposed to PPM, no chemical was detected in analyzed samples (Table 4).

## Discussion

In the framework of malaria prevention, chemical analysis of insecticide-treated materials used for mosquito control interventions and insecticide resistance testing (e.g., mosquito nets, filter papers) is strongly recommended for quality control [34]. In parallel, data on chemical uptake by mosquitoes through tarsal contact with insecticide-treated material may improve the monitoring of insecticide effects on mosquito populations. To ensure its functionality, the UHPLC-MS/MS method described here has been developed and validated using a reference laboratory colony of malaria vector species (KISUM1 *An*. *gambiae s*.*s*.), five well known insecticides used in malaria vector control (DM, PM, EPX, PPM and aCYP) and the WHO’s standard protocol for susceptibility test on adult mosquitoes [29].

Insecticide compounds were extracted using ultrasound treatment which allows the disintegration of cell walls via the formation of cavitation bubbles [35] and low mLOD and mLOQ were achieved without further sample treatment. Grinding with liquid nitrogen was considered impracticable due to the small sample size of mosquitoes (80–100 specimens). The limits of quantification were comparable to previously reported limits for LC-MS/MS in pesticide analysis of food matrices [18]. Assuming a sample size of 100 mg mosquito biomass or 100 individuals, a quantification limit of 1 μg L^-1^ would correspond to 10 ng per g mosquito biomass respective 10 pg per individual. As assay mosquito samples differed in both biomass and numbers of individuals, these specific quantification limits were not determined. For samples containing 1000 μg kg^-1^, the method precision is expected to be below 16% according to the Horwitz Equation [36]. Thus, precisions below 10% as obtained in most cases for the matrix samples here can be considered as acceptable. Assay derived variations (e.g. mosquito individual differences, actual DC on filter paper used for the test, individual contact time of mosquito with impregnated papers) were found to be much higher (see Discussion below). According to the guidance document for pesticide analysis in food and feed (SANTE/11813/2017 [25]), no adjustment of the analytical results has to be performed, when the recovery rate is between 80% and 120%. Consequently, only results for low concentrated PM samples were corrected by calculation. To enable comparability between surviving and dead mosquitoes, results of extracts showing a PM concentration close to the mLOQ were adjusted by factor 1.2 to simulate equal recovery rates for high and low concentrated extracts.

Within sample stability tests, an increase of the recovery rate was observed in some cases. This was most pronounced for PPM, the only compound without an isotopic labelled internal standard (Table 3). The increase might be due to reduced stability of PPE compared to PPM leading to higher recovery rates after storage. Additionally, the matrix might have changed during the storage, leading to a signal enhancement in case of PPM or suppression in case of PPE. Reduced stability of small concentrations was also observed in the case of EPX. As the deuterated compound was utilized as an internal standard, matrix changes should affect both analytes in a similar manner, and compared to PPM the increase of the recovery rate was less pronounced for EPX. The internal standard D_5_-EPX was present in a forty times higher concentration (0.5 vs. 20 μg L^-1^), so potential degradation processes might have followed (slightly) different kinetics. As for PPM, analyses of sample extracts should be performed right after preparation if concentrations below 2.5 μg L^-1^ are expected.

Bioassay data revealed a high knockdown effect of pyrethroids and pseudo-pyrethroid on the KISUMU1 strain of *An*. *gambiae s*.*s*. With PPM, the knockdown rates were very low because organophosphates do not act as fast as pyrethroids [37]. The mortality rates 24 hours post-exposure to insecticide-impregnated papers (99–100%) confirmed the susceptibility of this mosquito strain to insecticides in most of the assays, except two PM and three PPM assays. Since this mosquito strain is known to be free of any detectable insecticide resistance mechanism, the survival of mosquitoes during the above-mentioned assays may be related to the (in)stability of insecticides on impregnated papers over the time or to chemical exito-repellency (e.g. PM) rather than phenotypic resistance. Especially, results obtained for PPM during two different periods suggest instability of the chemical on the impregnated filter paper. Although PPM possesses the lowest mLOD of all analytes, it was not detectable in the samples of the second period (Tables 2 and 4). Within a preliminary test, mortality rate of 100% was obtained for the same filter paper. These circumstances would not be detectable by simple descriptive analysis (determination of mortality rate) and underline the necessity of an analytical tool for insecticide analyses in mosquito samples. The filter papers utilized in the test derived from the same batches, they were stored in the refrigerator (4°C) during the study period and used up to four times. According to the WHO’s protocol [29], the activity of insecticide on impregnated papers declines with the number of usages and the number of mosquitoes tested. An insecticide-impregnated paper should not be utilized more than six times which corresponds to the exposure of approximately 150 mosquitoes in a single vessel [29]. This insecticide resistance assessment protocol relies on the use of one insecticide dose (i.e. diagnostic concentration), one exposure time, and the record of mortality rates 24 h post exposure. However, the absence of mortality resulting from this method does not necessarily imply a complete absence of mortality due to insecticides. The phenotypic expression of resistance and the resistance level are highly dependent upon environmental variables like temperature [38], food quality/quantity [39], multiple blood meals [40] and preexisting pesticide exposure [41], all variations that are not captured in standardized bioassays. More interestingly, the current study revealed no significant decrease of weight in mosquitoes surviving after exposure to PPM and PM compared with control individuals, suggesting weight loss in dead samples as an indicator of insecticide activity in mosquito matrix. Insecticides can possess a dehydrating effect as it has been described for other insects and substances [42, 43].

In dead mosquito samples, the mean chemical uptake increased with the insecticide diagnostic concentrations from aCYP or DM (0.05%) to permethrin (0.75%). The highest mass was obtained with EPX although its diagnostic concentration (0.5%) was lower than that of PM. EPX is less polar than PM (octanol-water partition coefficient PM 6.1 and EPX 6.9, data are taken from [26]) and might be easier attached to the mosquito surface and be taken up by the organism leading to higher masses per individual. In contrast to pyrethroids, a remarkable lower uptake of EPX was observed after repeated usage of the same impregnated filter paper (1 to 4 times), but mortality rates of 100% were obtained in all cases. These different uptakes might be due to a reduced DC on the filter paper e.g., because of higher substance uptake within the first three usages, although a six-time usage would be in agreement with WHO’s recommendations [29].

Amounts of 23 to 52 pg aCYP and DM per mosquito were sufficient to kill the organism (Table 4). In the case of honey bees, the reported LD_50_ is more than 1000 or 450 times higher for aCYP or DM, respectively (Fig 1). However, the substance is usually applied topically in case of honey bees, and the incubation is performed for 48 hours. The weight of dead mosquitos was approx. 0.5 mg after exposure to insecticides, whereas 75 to 95 mg have been reported as the body weight of honey bees [44, 45]. In this context, the determined insecticide masses per mosquito appear realistic due to the remarkably smaller body weight of *An*. *gambiae s*.*s*.

With PM survivors, the chemical uptake was around 25% reduced compared with dead mosquitoes during the first and second trial (Table 4). It is noteworthy that in dead specimens a higher PM mass per mosquito was detected during the third trial; so the survival rates could not be attributed to a decreased DC on the filter paper as discussed for EPX and PPM. Mosquitoes must have picked up less of the insecticide, e.g. due to less contact to the filter paper surface resulting from exito-repellency of PM. In any mosquito population, some individuals are repelled or irritated easier than others. This behavior may allow them to escape from the lethal effect of insecticides. Indeed, pooling 20–25 mosquitoes per tube during bioassays may enhance their movements and limit their contact with the treated substrate, impeding the accuracy of resistance/susceptibility data. These findings underline the usefulness of the developed UHPLC-MS/MS method for investigating the uptake of insecticides by adult mosquitos.

## Conclusion

The UHPLC-MS/MS method described here has revealed the minimum uptake of each selected insecticide required to killed individual mosquitoes after tarsal contact with treated materials. Therefore, the standard bioassay protocols, while useful to monitor insecticide resistance or bio efficacy of treated material in field and laboratory studies, may not provide enough evidence for the failure in insecticide delivery from treated material to target mosquito populations. Further studies are needed to investigate the influence of mosquito sex, cuticle thickness and other insecticide resistance on the rate of insecticide uptake.
